# The genus *Andraca* (Lepidoptera, Endromidae) in China with descriptions of a new species

**DOI:** 10.3897/zookeys.127.928

**Published:** 2011-09-08

**Authors:** Xing Wang, Ling Zeng, Min Wang

**Affiliations:** 1 Department of Entomology, South China Agricultural University, Guangzhou, Guangdong 510640, P.R. China. Present address: Institute of Entomology, College of Biosafety Science and Technology, Hunan Agricultural University, Changsha 410128 Hunan, China; and Provincial Key Laboratory for Biology and Control of Plant Diseases and Insect Pests, Changsha 410128, Hunan, China; 2 Department of Entomology, South China Agricultural University, Guangzhou, Guangdong 510640, P.R. China

**Keywords:** Taxonomy, Lepidoptera, Endromidae, *Andraca*, new species, China

## Abstract

The six species of the genus *Andraca* Walker hitherto known from China are reviewed, and a new species, *Andraca gongshanensis*, **sp. n.**, described from Yunnan Province, China. Adults and male genitalia of all examined species are illustrated, together with a distributional map. A key to all seven Chinese *Andraca* species is provided. The types of the new species are deposited in SCAU (South China Agricultural University, Guangzhou, China) and HUNAU (Hunan Agricultural University, Changsha, China).

## Introduction

The genus *Andraca* was established by Walker (1865) with *Andraca bipunctata* Walker 1865 as its type-species, a species well known as one of the most serious pests of tea plants (Chu and Wang 1996). It was placed in family Bombycidae for over 150 years, but was recently transferred to family Endromidae based on the molecular study of Zwick et al. (2011). Kishida (1993) reported *Andraca theae* and *Andraca olivacea* from Taiwan. Chu and Wang (1993) recorded three *Andraca* species from China: *Andraca bipunctata* is widely distributed in central and southern China, *Andraca henosa* Chu & Wang, 1993 was listed from Yunnan, and *Andraca hedra* Chu & Wang, 1993 from Hainan and Fujian; in this paper, they also included *Andraca gracilis* Butler 1885, which is currently placed in the genus *Pseudandraca* Miyata, 1970. Yang (1995) added one species, *Andraca flavamaculata* Yang, 1995, to the Chinese *Andraca* fauna. Owada et al. (2002) reviewed three species of *Andraca* from Vietnam and provided a world checklist. Zolotuhin and Witt (2009) recorded five *Andraca* species from Vietnam, describing two new species, *Andraca stueningi* Zolotuhin & Witt, 2009 and *Andraca melli* Zolotuhin & Witt, 2009, and newly treating two taxa, *Andraca trilochoides roepkei* Bryk, 1944 and *Andraca olivacea olivacens* Mell, 1958, as subspecies of *Andraca trilochoides* Moore, 1865 and *Andraca olivacea* Matsumura, 1927 respectively. At present, the genus *Andraca* consists of eight species ranging from the Himalayas to Southeast Asia.

In the present paper, seven Chinese *Andraca* species are reviewed, including the description of one new species *Andraca gongshanensis*, sp. n. The early stages of *Andraca theae* (Matsumura 1909) are described in detail. A key to the seven Chinese *Andraca* species is provided.

## Materials and methods

Specimens of the new species were collected by light trap. The types of previously described species in the Natural History Museum, London, UK (BMNH) were examined. Other materials examined in this study are preserved in SCAU and HUNAU. Morphological terminology used in descriptions follows Lemaire and Minet (1999).

## Taxonomy

### Andraca

Walker, 1865

http://species-id.net/wiki/Andraca

Andraca  Walker, 1865, *List Specimens lepid. Insects Colln Br. Mus.*, 32: 581. (Type species: *Andraca bipunctata* Walker, 1865, *List Specimens lepid. Insects Colln Br. Mus.*, 32: 582, by monotype. Type locality: Hindostan, India.)
Pseudoeupterote  Shiraki, 1911, *Catalogue Insectorum Noxiorum Formosarum*: 48. (Type species: *Oreta theae* Matsumura, 1909, *Thousand Insects of Japan*, 1: 582, by monotype. Type locality: Formosa (=Taiwan)). Type-species designation by monotype.

#### Description.

Forewing weakly falcate. Ground color varying from shades of brown to sandy grey.

Male genitalia. Uncus apically single-pointed to weakly indented; gnathos with two long, basally broad, upcurved arms; valvae basally broad, sclerotized, long or medium length; aedeagus short with apex truncated, cornuti present or absent.

Female genitalia (*Andraca bipunctata*). Eighth segment curved deeply, ventral margin of ostium bursae extends posteriorly as a broad bilobed plate, ductus bursae sclerotized distal to mid-point, tapering to half width; distal half unsclerotized with slight torsion, corpus bursae lacking a signum.

#### Distribution.

Oriental Region, S & E Palaearctic.

#### Remarks.

*Andraca* species have sometimes been described in *Mustilia* (e.g., Chu and Wang 1993, 1996), and misidentification has also been frequent (Chu and Wang 1993, 1996). *Andraca* was considered to belong to ‘the *Mustilia* lineage’ of Prismostictinae Forbes, 1955 (Holloway 1987; Minet 1994; Lemaire and Minet 1999; Holloway et al. 2001). Our own unpublished work also shows that *Andraca* is close to *Mustilia* Walker, 1865 and *Mustilizans* Yang, 1995, based on phylogenetic analysis of mitochondrial and nuclear DNA sequences (*COI* + *18S* +*28S*) (Wang 2010).

Sevastopulo (1938) described the fully grown larvae of the type species. The larvae are gregarious, have short hairs covering the body, and are often heavily parasitized. Pupation is in a thin cocoon of brown silk spun among leaves.

#### Key to the Chinese Andraca species

**Table:** 

1	Apex of forewing falcate	2
–	Apex of forewing not falcate	5
2	Uncus broad, gnathos extremely swollen medially	*Andraca bipunctata*
–	Uncus narrow, gnathos not swollen	3
3	Apex of valva boot-shaped	*Andraca flavamaculata* comb. rev.
–	Apex of valva rounded or truncate	4
4	Apex of valva rounded, gnathos long	*Andraca olivacea*
–	Apex of valva truncate, gnathos short	*Andraca gongshanensis* sp. n.
5	Apex of valva bifurcate	*Andraca theae*
–	Apex of valva rounded	6
6	Gnathos not swollen	*Andraca apodecta*
–	Gnathos extremely medially swollen	*Andraca melli*

### Andraca bipunctata

Walker, l865

http://species-id.net/wiki/Andraca_bipunctata

Figs 1–A 2–A

Andraca bipunctata  Walker, 1865, *List Specimens lepid. Insects Colln Br. Mus.*, 32: 582. Type locality: Hindustan, India.
Andraca bipunctata  Walker, 1862: Chu& Wang, 1993, *Sinozoologia*, 10: 241.
Andraca henosa  Chu & Wang, 1993, *Sinozoologia*,10: 242. Type locality: Yunnan, China.
Andraca henosa  Chu & Wang: Chu & Wang, 1996, *Fauna Sinica Insecta*, 5: 55.

#### Description.

**Figure 1.**
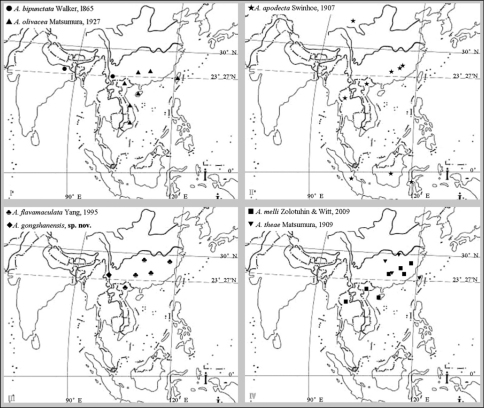
Distributional map of *Andraca* spp. from China.

**Figure 2.**
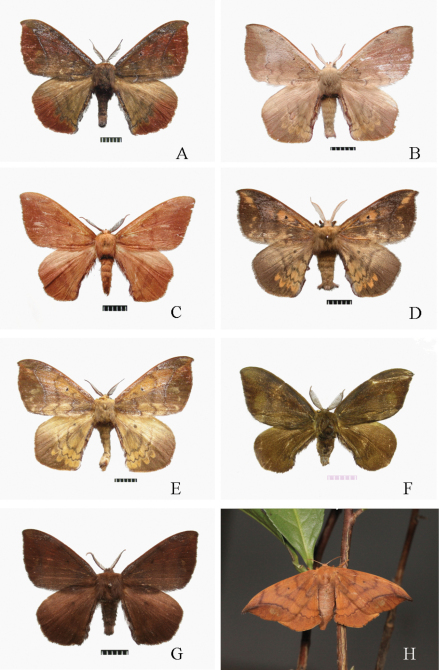
Adult male *Andraca* spp. **A** *Andraca bipunctata* Walker, l865 from Yunnan **B** *Andraca olivacea* Matsumura, 1927 from Guangdong **C** *Andraca apodecta* Swinhoe, 1907 from Guangxi **D** *Andraca flavamaculata* Yang, 1995 from Guangdong **E** *Andraca gongshanensis*, sp. n., Holotype, from Yunnan **F** *Andraca melli* Zolotuhin & Witt, 2009 from Guangdong **G** *Andraca theae* Matsumura, 1909 from Hunan Province **H** *Andraca theae* Matsumura, 1909 from Hunan Province (in field).

 Male (China): wingspan 42–45 mm, length of forewing 21–23 mm, antenna length 6–8 mm (Fig. 1-A). Male genitalia (Fig. 2-A): uncus broad, duck beak-shaped; gnathos long, finger-shaped; vesica with a cluster of strong spinose cornuti

Female genitalia: see above under generic entry.

#### Material Examined.

[CHINA]2♂♂, western Yunnan, 2005-VI-15, Ming-Yi Tian leg.; 2♂♂, Dulongjiang, Yunnan Province, 2006-VII-21, Min Wang & Xiao-Ling Fan leg.; 1♂1♀, Gongshan Mountain, Yunnan Province, 2006-VII-22 , Min Wang & Xiao-Ling Fan leg.

#### Host.

 *Camellia sinensis* (Theaceae), *Camellia Assamica* (Theaceae), *Camellia oleifera* (Theaceae).

#### Distribution.

 China (Yunnan); India.

#### Remarks.

This widely distributed species is rather variable in coloration and size. Moore (1865) described *Andraca trilochoides* from a brighter and grayish individual. This taxon was later synonymized with *Andraca bipunctata* by Hampson, ([1893]), an action that was followed by Strand (1924).

*Andraca bipunctata* is closely related to *Andraca angulata* Kishida, 1993 (Nepal and India: Sikkim), *Andraca theae* (Taiwan) and *Andraca stueningi* (Vietnam). These four species form the *bipunctata* group, and share the following characteristics: 1) male hindtibia with one pair of spurs; 2) two dorsally-directed projections present on subapical part of valva; 3) external surface of aedeagus partially covered with hair-like spines; 4) a cluster of strong spinose cornuti on vesica.

Larvae of *Andraca bipunctata* are well-known serious pests of tea trees, *Camellia sinensis* (Theaceae) (Banerjee 1982; Chang 1989; Chen et al. 1992; Panigrahi 1995; Ho et al. 1996; Upadhyay et al. 2001).

### Andraca olivacea

Matsumura, 1927

http://species-id.net/wiki/Andraca_olivacea

Figs 1–B 2–B

Andraca olivacea  Matsumura, 1927, *J. Coll. Agric. Hokkaido. Univ.*, 19: 50. Type locality: Formosa (=Taiwan), China.
Andraca hedra  Chu & Wang, 1993, *Sinozool*., 10: 233. Type locality: Hainan, China.
Andraca hedra  Chu & Wang: Chu & Wang, 1996, *Fauna Sinica Insecta*, 5: 58.
Andraca olivacea : Owada et al., 2002, *Spec. Bull. Jpn. Soc. Coleopterol*., 5: 464; Kishida, 1992, *Lepidoptera of Taiwan*, 1 (2): 153.

#### Description.

Male: wingspan 36–38 mm, length of forewing 16–20 mm, antenna length 5–7 mm (Fig. 1-B). Hindtibia with two pairs of spurs; hindwings with Rs and M1 connate. Male genitalia (Fig. 2-B): uncus thick and round; valva simple, basal half broad and terminal half narrow; distal margin of aedeagus with strong lateral spines; vesica with a cluster of spinose cornuti.

#### Material Examined.

[CHINA] 1 ♂, Shimentai Provincial Nature Reserve, Yingde City, Guangdong Province, 2001-VII-24, Min Wang & Guo-Hua Huang leg.; 1 ♂, same data but 2001-IX-22; 3 ♂ ♂, same data but 2002-VI-11, Guo-Hua Huang leg.; 1 ♂, Nanling National Nature Reserve, Ruyuan City, Guangdong Province, 2002-VII-23, Guo-Hua Huang leg.; 4 ♂ ♂, same data but 2003-III-29~31; 1 ♂, same data but 2003-VI-22; 2 ♂ ♂, same data but 2003-VIII-7; 2 ♂ ♂, same data but 2003-VIII-18; 5 ♂ ♂, same data but 2004-IV-23; 1 ♀, same data but 2004-IV-24; 2 ♂ ♂, same data but 2006-IX-18, Liu-Sheng Chen leg.; 1 ♂, same data but 2008-VI-7, Min Wang leg.; 1 ♂, same data but 2008-VI-7; 1 ♂, same data but 2009-IV-1, Hou-Shuai Wang leg.; 1 ♂ , same data but 2009-VIII-10; 1 ♂, same data but 2009-IV-1; 1 ♂ , same data but 2009-VIII-10; 1 ♂, Maoershan National Nature Reserve, Xingan City, Guangxi Province, 2003-III-3, Min Wang & Guo-Hua Huang leg.; 6 ♂ ♂, Jianfengling National Nature Reserve, Ledong City, Hainan Province, 2003-XI-29~31, Guo-Hua Huang & Min Wang leg.; 1 ♂, same data but 2007-X-23, Min Wang leg.

#### Host.

 *Ficus concinna* var. *pusillifolia* (Moraceae).

#### Distribution.

 China (Taiwan, Guangdong, Guangxi, Hainan); Vietnam.

#### Remarks.

Wang (1995) provide a fine color illustration of a fresh living male. Owada et al. (2002) considered *Andraca olivacens* from Fukien (= Fujian) to the synonym of *Andraca olivacea*, whereas Zolotuhin and Witt (2009) treated it as a subspecies thereof. We do not comment further on which of these two alternatives may be the most appropriate status for this taxon because we have not seen the types of *Andraca olivacens*.

### Andraca apodecta

Swinhoe, 1907

http://species-id.net/wiki/Andraca_apodecta

Figs 1–C 2–C

Andraca apodecta  Swinhoe, 1907, *Ann. Mag. nat. Hist.*, 19 (7): 49. Type locality: Sumatra, Indonesia.
Andraca apodecta  Swinhoe: Holloway, 1976, *Malayan Nature Society*: 85; Zolotuhin & Witt, 2009, *Entomofauna*, 261.

#### Description.

Male: wingspan 37–39 mm, length of forewing 15–18 mm, antenna length 6–8 mm (Fig. 1-C). Head covered with reddish-brown hairs; forewing with black discal spot, smooth outer margin and apically not falcate. Male genitalia (Fig. 2-C): uncus triangular, apical half truncate; valva with two subapical, dorsally-directed projections; aedeagus short, curved slightly without cornuti, external surface without hair-like spines.

#### Material Examined.

[CHINA]2♂♂, Jinzhongshan Mountain, Longlin City, Guangxi Province, 2007-VII-31, Liu-Sheng Chen leg..

#### Host.

 Unknown.

#### Distribution.

 China (Guangxi, Yunnan, Fujian, Shaanxi), Vietnam, Thailand (Chiang Mai, Nan), Indonesia (Sumatra, Borneo, Sulawesi).

#### Remarks.

The species was first recorded from China (Yunnan, Fujian, Shaanxi) by Zolotuhin and Witt (2009) and is here recorded from Guangxi for the first time.

### Andraca flavamaculata

Yang, 1995 comb. rev.

http://species-id.net/wiki/Andraca_flavamaculata

Figs 1–D 2–D

Andraca flavamaculata  Yang, 1995, *Insects of Baishanzu Mountain, Eastern China*: 354. Type locality: Zhejiang, China.
Andraca nabesan  Kishida & Owada, 2002, *Spec. Bull. Jpn. Soc. Coleopterol.*, (5): 464; Huang & Wang, 2004, *Entomotaxonomia*, 26(1): 47. Type locality: Cao Bang, Vietnam.

#### Description.

Male: wingspan 40–44 mm, length of forewing 20–22 mm, antenna length 6–7 mm (Fig. 1-D). Body stout. Forewing apex falcate; outer edge smooth and straight; tornus almost rectangular. Male genitalia (Fig. 2-D): uncus long with apex finger-shaped; tegumen broad with numerous long setae; valvae basally broad, strongly sclerotized, apex of valva boot-shaped; sacculus broad, with a strong dorsal spike; saccus short and narrow; aedeagus short but strong and straight, distally with a large number of spines.

#### Material Examined.

 [CHINA] 2 ♂♂, Nanling National Nature Reserve, Ruyuan City, Guangdong Province, 2002-III-15, Guo-Hua Huang leg.; 2 ♂♂, same data but 2003-II-23; 5 ♂ ♂, same data but 2003-III-29 ~ 31; 1 ♂, same data but 2003 -VIII-30; 1 ♂, same data but 2006-IX-17, Zhen Li leg.; 2 ♂♂ , Maoershan National Nature Reserve, Xingan City, Guangxi Province, 2003-III-03, Min Wang & Guo-Hua Huang leg.; 3 ♂ ♂, Mangshan Nature Reserve, Yizhang City, Hunan Province, 2003-III-31, Guo-Hua Huang leg.; 1 ♂, Jiuwandashan National Nature Reserve, Guangxi Province, 2003-VII-30, Guo-Hua Huang leg.

#### Host.

 Unknown.

#### Distribution.

 China (Zhejiang, Hunan, Guangdong, Guangxi); Vietnam.

#### Remarks.

 Yang (1995) described the species from Zhejiang, China. The species is similarto *Andraca olivacea* but can be distinguished by the following characters: aedeagus straight; gnathos not prominent. Zolotuhin and Witt (2009) synonymized *Andraca nabesan* Kishida & Owada, 2002 with *Andraca flavamaculata*, which they also transferred to *Pseudandraca* species based on features of the genitalia. We accept the synonymy but do not agree with the generic transfer, because we do not consider that the diagnostic feature of *Pseudandraca* given by Miyata (1970), a valva with a “long distinct projection” is present in *Andraca flavamaculata*. We therefore transfer *Andraca flavamaculata* comb. rev. back to *Andraca*.

### Andraca gongshanensis sp. n.

urn:lsid:zoobank.org:act:E5DD5FB7-554B-48A6-9EF3-65F1699E9897

http://species-id.net/wiki/Andraca_gongshanensis

Figs 1–E 2–E 

#### Description.

Male: wingspan 46–48 mm, length of forewing 22–24 mm, antenna length 5–8 mm (Fig. 1-E). Antenna bipectinate except apex. Wings ground color dark brow with dark brow fasciae and reddish-yellow patterns, which is consisting of antemedian, discocellar, postmedian fascia, and reddish-yellow patterns nearly placed on the wholly wings but termen. Forewing apex falcate; outer edge smooth and straight; tornus almost rectangular. Hindwing with anal margin straight; outer margin angled at vein M3, straight above and below this.

Male genitalia (Fig. 2-E): uncus long with wedge-shaped apex; tegumen broad; gnathos very well developed, arms upcurved; valvae basally broad with many long setae, strongly sclerotized, caudally constricted to a spatulate apex; sacculus broad, without a dorsal spike; aedeagus short but strong and straight, distally with a large number of spines.

#### Holotype.

 ♂, Gongshan Mountain, Yunnan Province, China, 2006-VII-22, Min Wang & Xiao-Ling Fan leg., deposited in Department of Entomology, SCAU; **Paratypes**, 2 ♂♂, same data as holotype but 2006-VII-21.; 1 ♂, same data as holotype but 2006-VII-23; deposited in Institute of Entomology, HUNAU.

#### Host.

 Unknown.

#### Distribution.

 China (Yunnan).

#### Etymology.

 The specific epithet refers to the type locality(Gongshan Mountain, China).

#### Remarks.

This new species is very similar to *Andraca flavamaculata*, but can be distinguished by the following characters of the male genitalia: *Andraca gongshanensis*, sp. n. with uncus apex wedge-shaped,apex of valva constricted and truncate, sacculus without a strong dorsal spike. And *Andraca flavamaculata* with uncus apex finger-shaped, apex of valva boot-shaped; sacculus broad, with a strong dorsal spike.

Externally, *Andraca gongshanensis* is paler than *Andraca flavamaculata*.

### Andraca melli

Zolotuhin & Witt, 2009

http://species-id.net/wiki/Andraca_melli

Figs 1–F 2–F

Andraca melli  Zolotuhin & Witt, 2009, *Entomofauna*, Suppl. 16: 262. Type locality: Guangdong, China.

#### Description.

Male: wingspan 37–39 mm, length of forewing 15–18 mm, antenna length 5–7 mm (Fig. 1-F). Antenna bipectinate except apex. Head thinly covered with brown-green hairs. Forewing: apically bluntly pointed; outer edge smooth and straight, tornus nearly rectangular. Hindwings distinctly angled at vein M3, straight above and below this.

Male genitalia (Fig. 2-F): uncus bluntly triangular with long hairs; tegumen broad; gnathos with two extremely medially swollen arms; valvae flattened, strongly sclerotized, apex narrower and truncate with a dorsally directed projection from the middle; saccus short and broad; aedeagus short, strongly curved, with a compact group of long, thick needle-shaped cornuti on dorsal surface.

#### Material Examined.

 [CHINA]2 ♂♂, Nanling National Nature Reserve, Ruyuan City, Guangdong Province, 2007-VI-23, Liu-Sheng Chen collected larvae and reared to adult.

#### Host.

 *Camellia sinensis* (Theaceae), *Camellia oleifera* (Theaceae), *Fraxinus pennsylvanica* (Oleaceae) and *Ternstroemia japonica* (Ternstroemiaceae), *Pentaphylax euryoides* Gardn. & Champ. (Pentaphylacaceae) (new host record).

#### Distribution.

China (Zhejiang, Jiangxi, Fujian, Guangdong, Hainan); Vietnam; Thailand.

#### Remarks.

*Andraca melli* was first described by Zolotuhin and Witt (2009), who also reported on the biology of this species.

### Andraca theae

Matsumura, 1909

http://species-id.net/wiki/Andraca_theae

Figs 1–G, 1–H 2–G, 2–H

Oreta theae  Matsumura, 1909, *Thousand Insects of Japan*, 1: 86. Type locality: Formosa (= Taiwan), China.

#### Description.

Male: wingspan 35–37 mm, length of forewing 17–19 mm, antenna length 6–7 mm (Figs 1-G, 1-H). Head densely covered with dark brown hairs; antenna bipectinate except apex. Forewing apex inconspicuously falcate, exterior margin straight. Forewing and hindwing each with a dark discal spot.

Male genitalia (Fig. 2-G): uncus triangular with apex narrowly spatulate; tegumen broad; gnathos elongate, medially inflated; saccus short and broad; valvae bifurcate apically; dorsal margin with a subapical hump; aedeagus bowed with dense apical spines.

#### Material Examined.

[CHINA] 1 ♂, Nanling National Nature Reserve, Ruyuan City, Guangdong Province, 2003-III-29, Guo-Hua Huang leg.; 1 ♂, same data to the former, except 2003-VIII-12, Guo-Hua Huang & De-Yu Xin leg.; 2 ♂♂, Taibei City, Taiwan Province, 2009-VIII-15, Shipher Wu leg.; 10 ♂♂, Wuyunjie National Nature Reserve, Taoyuan City, Hunan Province, 2010-VII-2, collected the larvae in the field by Mr. Hong-Chun Zhou, got the adults from the larvae bred in the entomological laboratory of Hunan Agricultural University by Dr. Guo-Hua Huang; 3 ♂♂, Houxi Town, Huangshan City, Anhui Province, 2010-VI-28, the adults from the larvae collected in the field and bred in laboratory by Dr. Guo-Hua Huang.

#### Host.

 *Camellia sinensis* (Theaceae).

#### Biology.

**Figure 3.**
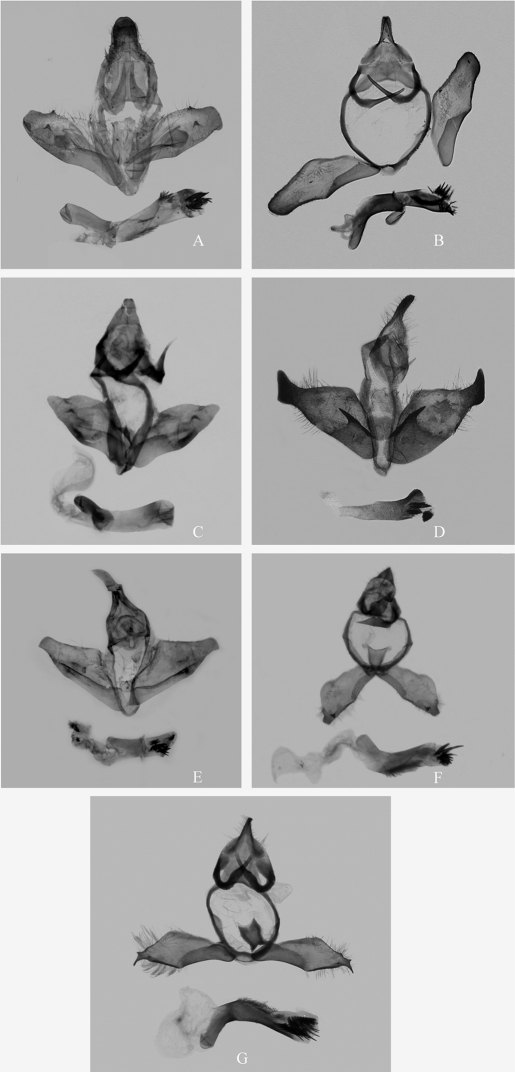
Male genitalia of Chinese *Andraca* spp. **A** *Andraca bipunctata* Walker, l865 from Yunnan **B** *Andraca olivacea* Matsumura, 1927 from Guangdong **C** *Andraca apodecta* Swinhoe, 1907 from Guangxi **D** *Andraca flavamaculata* Yang, 1995 from Guangdong **E** *Andraca gongshanensis*, **sp. n.**, Holotype, from Yunnan **F** *Andraca melli* Zolotuhin & Witt, 2009 from Guangdong **G** *Andraca theae* Matsumura, 1909 from Hunan Province.

 This species is widely distributed in Taiwan and Southern China. The larvae were found on *Camellia sinensis* in Hunan Province; photographs of the early stages were taken in June to August, 2010 (Figs 3-A to 3-H).

#### Distribution.

 China (Taiwan, Guangdong, Hunan, Anhui).

#### Remarks.

 The species is easily separated from its congeners by the apically bifurcate valvae.

**Figure 4.**
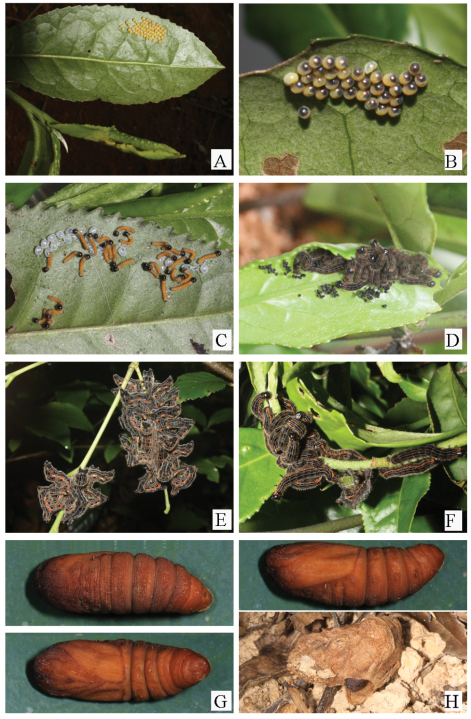
The early stages of *Andraca theae* Matsumura, 1909 from Hunan Province **A–B** Eggs **C** First larvae **D** Third larvae **E–F** Final larvae **G–H** Pupa and cocoon.

## Supplementary Material

XML Treatment for Andraca

XML Treatment for Andraca bipunctata

XML Treatment for Andraca olivacea

XML Treatment for Andraca apodecta

XML Treatment for Andraca flavamaculata

XML Treatment for Andraca gongshanensis

XML Treatment for Andraca melli

XML Treatment for Andraca theae

## References

[B1] Banerjee B (1982) A strategy for the control of *Andraca bipunctata* Walker on tea. Crop Protection 1 (1): 115-119

[B2] Bryk F (1945) Entomological results from the Swedish expedition 1934 to Burma and British India. Lepidoptera: Saturniidae, Bombycidae, Eupterotidae, Uraniidae, Epiplemidae und Sphingidae. Arkiv Zoology 35A (8): 1–55, pls. 1–6.

[B3] Chang HG (1989) Effects of temperature on development of the egg stage of the tea bunch caterpillar *Andraca bipunctata* Walker. Journal of Anhui Agricultural College 16 (2): 137-140

[B4] Chen JX Ding YG Xu DJ (1992) Studies on a granulosis virus of the tea bunch caterpillar *Andraca bipunctata* [LEP.: Bombycidae] and its utilization. Chinese Journal of Biological Control 8 (2): 72-76

[B5] Chu HF Wang LY (1993) Saturniidae of China. Bulletin of Zoology. Science Press. Beijing, 10: 211-238

[B6] Chu HF Wang LY (1996) Fauna Sinica Insecta V, Lepidoptera: Bombycidae. Science Press Beijing, 1996: 24pp.

[B7] Davis DR (1992) Bombycidae. In: Heppner JB Inoue H (Eds). Lepidoptera of Taiwan 1 (2): Checklist.Scientific Publishers, Gainesville/Washington/Hamburg/Lima/Taipei/ Tokyo: 153-154

[B8] Hampson GF ([1893]) Moths, 1. In: Fauna of British India, Including Ceylon and Burma. Taylor & Francis, London xxiii + 527 pp.

[B9] Ho HY Tao YT Tsai RS Wu YL Tseng HK Chow YS (1996) Isolation, identification, and synthesis of sex pheromone components of female tea cluster caterpillar *Andraca bipunctata* walker (Lepidoptera: Bombycidae) in Taiwan. Journal of Chemical Ecology 22 (2): 271-285 10.1007/BF02055098 24227409

[B10] Holloway JD (1976) Moths of Borneo with Special Reference to Mount Kinabalu. Malayan Nature Society, Kuala Lumpur [viii] + 264 pp.

[B11] Holloway JD (1987) The moths of Borneo (III). Malaysian Nature Society, Kuala Lumpur, 74–90

[B12] Holloway JD Kibby G Peggie D (2001) Fauna Malesiana Handbook, 3. The Families of Malesian Moths and Butterflies. Brill, Leiden/Boston/Köln xi + 455 pp.

[B13] Kishida Y (1993) Bombycidae. In: Haruta T (Ed) Moths of Nepal, Part 2. Tinea 13 (Supplement 3): 143–145, pl. 57.

[B14] Lemaire C Minet J (1999) The Bombycoidea and their relatives. In: Kristensen NP (Ed). Lepidoptera, Moths and Butterflies.1. Evolution, systematics and biogeography. Handbook of Zoology 4(35), Walter de Gruyter, Berlin & New York: 321-354

[B15] Minet J (1994) The Bombycoidea–Phylogeny and higher classification (Lepidoptera, Glossata). Entomologica Scandinavica 25 (1): 63-88

[B16] Miyata T (1970) A generic revision of the Japanese Bombycidae, with description of a new genus (Lepidoptera). Tinea 8: 190-199

[B17] Moore F (1865) On the lepidopterous insects of Bengal. Proceedings of the Zoological Society of London, 1865: 755–823, pls. 41–43

[B18] Owada M Kishida Y Thinh TH Jinbo U (2002) Moths of the genus *Andraca* (Lepidoptera, Bombycidae, Prismostictinae) from Vietnam. Special Bulletin of the Japanese Society of Coleopterology (5): 461–472

[B19] Panigrahi A (1995) Fungus *Cordyceps militaris* (Fries) link infestation in the pupa of the tea pest *Andraca bipunctata* Walker. Environment and Ecology 13 (4): 942-946

[B20] Sevastopulo DG (1938) Early stages of Indian Lepidoptera (18). Journal of Bombay Natural History Society 35: 40-46

[B21] Strand E (1924) Bombycidae. In: Seitz A (Ed) Macrolepidoptera of the World 10: 436–442, 457

[B22] Upadhyay RK Mukerji KG Chamola BP (Editor) (2001) Biocontrol Potential and its Exploitation in Sustainable Agriculture: Volume 2: Insect Pests. Springer Publisher, 162–163

[B23] Walker F (1865) List of the specimens of lepidopterous insects in the collection of the British Museum 32, (suppl. to part 2), London, 323–706

[B24] Wang HY (1995) Guide Book to Insects in Taiwan: Bombycidae, Thyatiridae, Limacodidae, Lasiocampidae and Sphingidae. Shuhsing Publishing, Taipei, 10–11

[B25] Wang X (2010) Studies on the Systematics of Bombycidae from China (Lepidoptera: Bombycoidea). Unpublished PhD Thesis (South China Agricultural University, China)

[B26] Yang JK (1995) Lepidoptera: Bombycidae. In: Wu H (Ed). East Hill Zukun hundred insects.China Forestry Publishing House, Beijing: 353-358

[B27] Zolotuhin V Witt TJ (2009) The Bombycidae of Vietnam. Entomofauna, (Suppl. 16): 231–272

[B28] Zwick A Regier JC Mitter C Cummings MP (2011) Increased gene sampling yields robust support for higher-level clades within Bombycoidea (Lepidoptera). Systematic Entomology, 36: 31-43

